# Promoting healthy teenage behaviour across three European countries through the use of a novel smartphone technology platform, PEGASO fit for future: study protocol of a quasi-experimental, controlled, multi-Centre trial

**DOI:** 10.1186/s12911-019-0958-x

**Published:** 2019-12-17

**Authors:** Elisa Puigdomenech, Anne Martin, Alexandra Lang, Fulvio Adorni, Santiago Felipe Gomez, Brian McKinstry, Federica Prinelli, Laura Condon, Rajeeb Rashid, Maurizio Caon, Sarah Atkinson, Claudio L. Lafortuna, Valentina Ciociola, Janet Hanley, Lucy McCloughan, Conxa Castell, Mireia Espallargues, on behalf of the PEGASO Fit For Future Consortium, Carme Carrion, Mireia Espallargues, Santiago Felipe Gomez, Elisa Puigdomenech, Conxa Castell, Till Becker, Ian Dunwell, Kim Bul, Fulvio Adorni, Martina Camarenti, Valentina Ciociola, Chiara Crespi, Nithiya Jesuthasan, Claudio Lafortuna, Gianfranco Modoni, Federica Prinelli, Giovanna Rizzo, Marco Sacco, Aleksandra Sojic, Sarah Tabozzi, Nithiya Jesuthasan, Olivier Grossenbacher, Mathieu Lemay, Enric Muntané Calvo, Felip Miralles, Silvia Orte, Marc Solà, Filip Velickovski, Mauro Brivio, Maria Renata Guarneri, Leonardo Angelini, Maurizio Caon, Stefano Carrino, Elena Mugellini, Cesare Delaini, Dalia Morosini, Marco Decandia, Sara Facchinetti, Andrea Migliavacca, Silvana Mura, Luca Bianchi, Marco Mazzola, Sandro Repetti, Giuseppe Andreoni, Alessandra Mazzola, Paolo Perego, Carlo Emilio Standoli, Ciprian Candea, Gabriela Candea, Massimiliano Azzolini, Luca Bianconi, Marco Costacurta, Cristiana Degano, Fabio Podda, Antonio Ascolese, Lucia Pannese, Lucy McCloughan, Janet Hanley, Yvonne Laird, Anne Martin, Brian McKinstry, Rajeeb Rashid, George Scott, Jose Serrano, Sarah Atkinson, Sue Cobb, Laura Condon, Neil Coulson, Alexandra Lang, Alyson Langley

**Affiliations:** 1 Agency for Health Quality and Assessment of Catalonia (AQuAS), Catalan Department of Health, Roc Boronat 81-95, 2nd floor, 08005 Barcelona, Spain; 2 Health Services Research on Chronic Patients Network (REDISSEC), Instituto de Salud Carlos III, Roc Boronat 81-95, 2nd floor, 08005 Barcelona, Spain; 3 MRC/CSO Social and Public Health Sciences Unit, University of Glasgow, 200 Renfield Street, G2 3AX Glasgow, UK; 4 Human Factors Research Group, Faculty of Engineering, University of Nottingham, University Park, NG7 2RD Nottingham, UK; 5 National Research Council, Institute of Biomedical Technologies, Via Fratelli Cervi, 93, 20090 Segrate (MI), Italy; 6 Programs Department Gasol Foundation, 26-28 Jaume I street, 08830 Sant Boi de Llobregat, Spain; 7 GREpS. Health Education Research Group,Nursing and Phisiotherapy Department, University of Lleida, 2 Montserrat Roig street, 25198 Lleida, Spain; 8 Usher Institute,University of Edinburgh, University of Edinburgh, NINE Edinburgh BioQuarter, 9 Little France Road, EH16 4UX Edinburgh, UK; 9 National Research Council, Institute of Biomedical Technologies, Via Fratelli Cervi, 93, 20090 Segrate (MI), Italy; 10 PRISM Research Group, Division of Primary Care, School of Medicine, University of Nottingham, Room 1404, Tower Building, University Park, NG7 2RD Nottingham, UK; 11 Deanery of Clinical Sciences, College of Medicine and Veterinary Medicine, The Queen’s Medical Research Institute, University of Edinburgh, Edinburgh BioQuarter, 47 Little France Crescent, EH16 4TJ Edinburgh, UK; 12 College of Engineering & School of Management, University of Applied Sciences and Arts Western Switzerland (HES-SO), chemin du musée 4n, 1700 Fribourg, Switzerland; 13 Human Factors Research Group, Faculty of Engineering, The University of Nottingham, University Park, NG7 2RD Nottingham, UK; 14 Consiglio Nazionale delle Ricerche, Istituto di Fisiologia Clinica, Piazza Ospedale Maggiore, 3, 20162 Milano, Italy; 15 National Research Council, Institute of Biomedical Technologies, Via Fratelli Cervi, 93, 20090 Segrate (MI), Italy; 16 School of Health and Social Care, Edinburgh Napier University, Sighthill Court, EH41 3ND Edinburgh, UK; 17 Usher Institute, University of Edinburgh, NINE Edinburgh BioQuarter, 9 Little France Road, EH16 4UX Edinburgh, UK; 18 Catalonia Public Health Agency, Catalan Department of Health, Roc Boronat 81-95, 3rd floor, 08005 Barcelona, Spain

**Keywords:** Adolescents, Obesity prevention, Behaviour change, Physical activity, Diet, Sleep, Sedentary behaviour, Health promotion, Mobile health, mHealth, eHealth, Smartphone application, Serious game

## Abstract

**Background:**

Behaviour change interventions targeting physical activity, diet, sleep and sedentary behaviour of teenagers show promise when delivered through smartphones. However, to date there is no evidence of effectiveness of multicomponent smartphone-based interventions. Utilising a user-centred design approach, we developed a theory-based, multi-dimensional system, PEGASO Fit For Future (PEGASO F4F), which exploits sophisticated game mechanics involving smartphone applications, a smartphone game and activity sensors to motivate teenagers to take an active role in adopting and maintaining a healthy lifestyle. This paper describes the study protocol to assess the feasibility, usability and effectiveness (knowledge/awareness and behavioural change in lifestyle) of the PEGASO system.

**Methods:**

We are conducting a quasi-experimental controlled cluster trial in 4 sites in Spain, Italy, and UK (England, Scotland) over 6 months. We plan to recruit 525, in a 2:1 basis, teenagers aged 13–16 years from secondary schools. The intervention group is provided with the PEGASO system whereas the comparison group continues their usual educational routine. Outcomes include feasibility, acceptance, and usability of the PEGASO system as well as between and within group changes in motivation, self-reported diet, physical activity, sedentary and sleeping behaviour, anthropometric measures and knowledge about a healthy lifestyle.

**Discussion:**

PEGASO F4F will provide evidence into the cross-cultural similarities and differences in the feasibility, acceptability and usability of a multi-dimensional smartphone based behaviour change intervention for teenagers. The study will explore facilitating factors, challenges and barriers of engaging teenagers to adapt and maintain a healthy lifestyle when using smartphone technology. Positive results from this ICT based multi component intervention may have significant implications both at clinical level, improving teenagers health and at public health level since it can present an influential tool against the development of chronic disease during adulthood.

**Trial registration:**

https://clinicaltrials.gov Registration number: NCT02930148, registered 4 October 2016.

## Background

Adolescence is a period characterised by significant changes at physical and psychological levels [1]. It is a life-course stage vulnerable to acquiring unhealthy behaviours such as insufficient levels of physical activity and excess of sedentary behaviour, poor diet and sleep deprivation [2]. Several studies highlight the importance of adopting and maintaining healthy lifestyles amongst young people to ensure physical health and mental wellbeing during adolescence and adulthood [3–6]. Interventions that facilitate healthy dietary habits [7]; regular physical activity [8]; adequate sleep duration [9], and reduced sedentary behaviour [10, 11] were shown to be effective in preventing and treating obesity and other risk factors of non-communicable conditions such as type II diabetes, cardiovascular disease or cancer. However, available evidence is still overall considered to be of low to moderate quality [12, 13].

In Europe the proportion of overweight individuals in the general population is a relevant public health problem. According to the World Health Organization (WHO), the prevalence of overweight (body mass index >85th percentile) among European 13-year-olds is 27% [6, 14]. Overweight during childhood and adolescence is a risk factor for obesity in adulthood [15] and thus overweight control by empowering healthy lifestyles habits among adolescents is generally considered a fruitful strategy of public health promotion. Several social, school-based and media campaigns have been conducted aiming to motivate behaviour change [12]. However, in children and adolescents, evidence of a reduction in overweight and obesity prevalence is lacking [16] and the prevalence of related unhealthy behaviour is still very high [17]. This suggests that innovative intervention strategies, more engaging and attractive for children and adolescents, are needed.

In Europe, as well as in other regions, the development of electronic and mobile Health (eHealth and mHealth) has largely focused on the management of chronic diseases [18–21]. The combination of these and further integrated health applications (apps) is referred to as Health 4.0, which is the ultimate goal of ubiquitous, intelligent and interactive health service provision [22]. Nowadays, adolescents live in a highly technological world rapidly adopting information and communication technologies (ICTs) in general, and especially mobile devices such as smartphones, tablet computers [23] and electronic bracelets [24]. Digital platforms allow adolescents to go beyond face-to-face interaction, to be connected online and to have access to information from social media and networks, independently from the socioeconomic or familiar strata [25]. In this context, public health authorities have an opportunity to disseminate key messages to adolescents to promote healthy decision-making and lifestyles in a creative and innovative way relevant to the intended user population [26].

Recent reviews show that besides being feasible and acceptable, the use of mobile and wireless technologies in adolescents can help to prevent and treat overweight condition and obesity among children and adolescents [27–29]. The literature review undertaken by Dute and collaborators on mHealth based interventions to promote healthy habits among adolescents, showed that ‘only 4 apps were developed specifically for adolescents. All apps were tested on a small scale and for a short period’ [29]. Mobile apps for promoting healthy eating and physical activity tend to provide the user with the opportunity to set personal goals, to self-monitor their healthy behaviour and to receive tailored feedback. Although known to be effective, other behaviour change techniques such as social support are rarely present in health promotion apps for adolescents to date [29].

This paper presents the study protocol of a cross-cultural mHealth behaviour change intervention for use by teenagers, PEGASO Fit For Future (PEGASO F4F), designed to provide proactive health promotion in a real-world environment for overweight and obesity prevention. The study aims to understand and test the acceptance of a suite of integrated technologies (PEGASO system), the user experience and the effects of the PEGASO system on:

- change of knowledge about healthy eating, physical activity, sitting (sedentary behaviour) and sleeping habits
- motivation to change obesity-related lifestyle behaviours
- behavioural change in obesity-related lifestyle behaviours

## Methods

### Design and settings

A quasi-experimental cluster controlled trial was undertaken to evaluate an ICT-based intervention over 6 months, i.e., the PEGASO System, to promote healthy habits among adolescents. This study includes adolescents of 12 schools from four sites: England (UK), Scotland (UK), Lombardy (Italy) and Catalonia (Spain).

The target sample size of 525 with 2:1 allocation (intervention: control) was designed to be large enough in each school to be reasonably sure that large scale recruitment was possible, to have a representation from different socioeconomic groups, to allow for attrition and to give some indication of variance in the various proposed outcome measures which would permit a sample size calculation for a future cluster randomized controlled trial.

Ethical approval was granted by the clinical research ethics committee (CEIC) of all four intervention sites: South East Scotland Research Ethics Committee in the UK, Istituto di Ricovero e Cura a Carattere Scientifico (IRCCS) Policlinico of Milan in Italy and CEIC of Institut d’Investigació en Atenció Primaria de Salut Jordi Gol (IDIAP Jordi Gol) in Spain.

### Recruitment and group allocation

Potential participants were identified in schools, which were approached through researcher’s contacts. The selection of schools was determined by our aim to include students from a range of socioeconomic backgrounds. Different recruitment strategies were applied in the schools. In two sites, whole classes were recruited to the intervention group and whole classes to the control group (from the same school or from a different one). Another recruitment strategy was that the intervention was offered to all students in eligible year groups and volunteers were sought with the first responders being chosen. Participants for the control group were similarly chosen from another school. Participant recruitment took place from September to November 2016.

### Participant selection criteria

Eligible for inclusion in this study were teenagers aged 13 to 16 years (inclusive) without physical or psychological conditions that would prevent the understanding of and their participation in the intervention, who were able to participate in the study for 6 months and had an adequate proficiency in the local language (Italian, Catalan/Spanish and English). Although it is generally accepted that adolescence extends beyond this age, range 13–16 years was chosen as the most feasible age range to introduce this type of intervention in a school context.

### Intervention

The PEGASO system is based on the comprehensive Behaviour Change Wheel (BCW) framework and the Capability-Opportunity-Motivation-Behaviour (COM-B) system [30]. This provides the theoretical grounding for the content and implementation approach of the behaviour change interventions embedded within the components of the PEGASO system (Table 1).

**Table 1:** Behaviour change technique (BCT) within each PEGASO system component

Behaviour change technique	Operationalisation in the PEGASO system
Companion App	
Information about health consequences, Prompt/Cues, Instruction on how to perform a behaviour	News stream UI
Feedback on behaviour	Energy bar
Self-monitoring of behaviour	Interaction with specific UI elements in the News Stream
Social support	Friends UI
Problem solving	Messages and reminders
EDiary App	
Self-monitoring of behaviour, Feedback on behaviour	Diversity and Balance index and Food groups consumption
Prompt/Cues	Current target behaviour
Credible source	Companion Avatar
Instruction on how to perform a behaviour	Companion Avatar suggestions
Challenges App	
Feedback on behaviour	Feedback on the performance in the challenge
Prompt/Cues	New challenge available
Self-monitoring of behaviour	Challenge status
Instruction on how to perform a behaviour	Challenge description
Social support	Competitive and collaborative challenges
Goal setting (behaviour)	Selection of challenge
Goal setting (outcome)	Selection of challenge
Dashboard App	
Self-monitoring of behaviour, Feedback on behaviour	Presentation of performances in activities related to physical activity
Serious Game	
Information about nutrients in food	Mini-games (Scavenge mini-game, Research mini-game, NPC mini-game, Laptop mini-game)
Feedback on behaviour	Energy bar, score and points, mini-games, game results
Feedback on the outcomes of the behaviour	Energy bar metaphor
Credible source	Game narrative
Adding objects to the environment	Game narrative
PEGASO Web Portal	
Information about health consequences	Blog information and training modules
Self-monitoring of behaviour Feedback on behaviour	ZivaCare module
Feedback on the outcomes of the behaviour	Blog information contact with PEGASO experts and training modules
Credible source	Contact with experts
Instruction on how to perform a behaviour	Blog information and training modules
Social support	Contact with peers, tutors and expert
Report App	
Self-monitoring of behaviour Feedback on behaviour	Display of stats about user behaviours
Social support	Provision of the possibility to interact with the family doctor

*UI*: User interaction

The innovative key component of the PEGASO system is the behaviour recognition system, which allows detection and evaluation of participants’ real-time behaviour (collected through other components of the PEGASO system). A summary of the obesity-related behaviours targeted by the PEGASO system is shown in Table 2.

**Table 2:** Target behaviours of the PEGASO system and behavioural goals

Target behaviour	Behavioural goals
Dietary target behaviours	- Fruit consumption of ≥2 servings (250-375 g)/day
	- Vegetable consumption of ≥2 servings (300-450 g)/day
	- Reduced intake of sugar-sweetened beverages
	- Daily breakfast consumption
	- Reduced intake of fast food
	- Reduced consumption of sweet and salty high-energy snack high-energy
Physical activity target behaviours	- 60 min of daily physical activity of moderate-to-vigorous intensity (i.e. involving energy expenditure over 4 METs)
	- Undertake 12.000 steps/day
	- Daily active transport to and from school
Sedentary behaviour	- less than 45% of after-school time daily spent in sedentary activities (≤1.5 METs)
Sleep behaviour	-Daily sleep duration of at least 8 h

*METs* Metabolic Equivalents

Participants have the opportunity to choose one target behaviour at the time. When selecting a new target behaviour, participants are asked to complete the COM-B questionnaire [30]. The answers for each question subset guide which component of the COM-B behaviour system is adopted to target the content of the behaviour change intervention. For instance, the messaging framework in the PEGASO Companion App selects messages that are associated with participants’ preferences expressed in the COM-B questionnaire. This provides an intervention that is personalised to the different needs of the individual intervention participant.

The PEGASO system was developed using a user-centred design approach aiming to deliver a product and interactions which are attractive to adolescents and which enabled them to connect with their peers.

### Components of the PEGASO platform

The PEGASO system includes physical activity monitoring devices, a web portal, and up to six apps: Companion app, Challenges app, EDiary app, Dashboard app, serious game and Report app.

#### Physical activity monitors

**Fig. 1:**
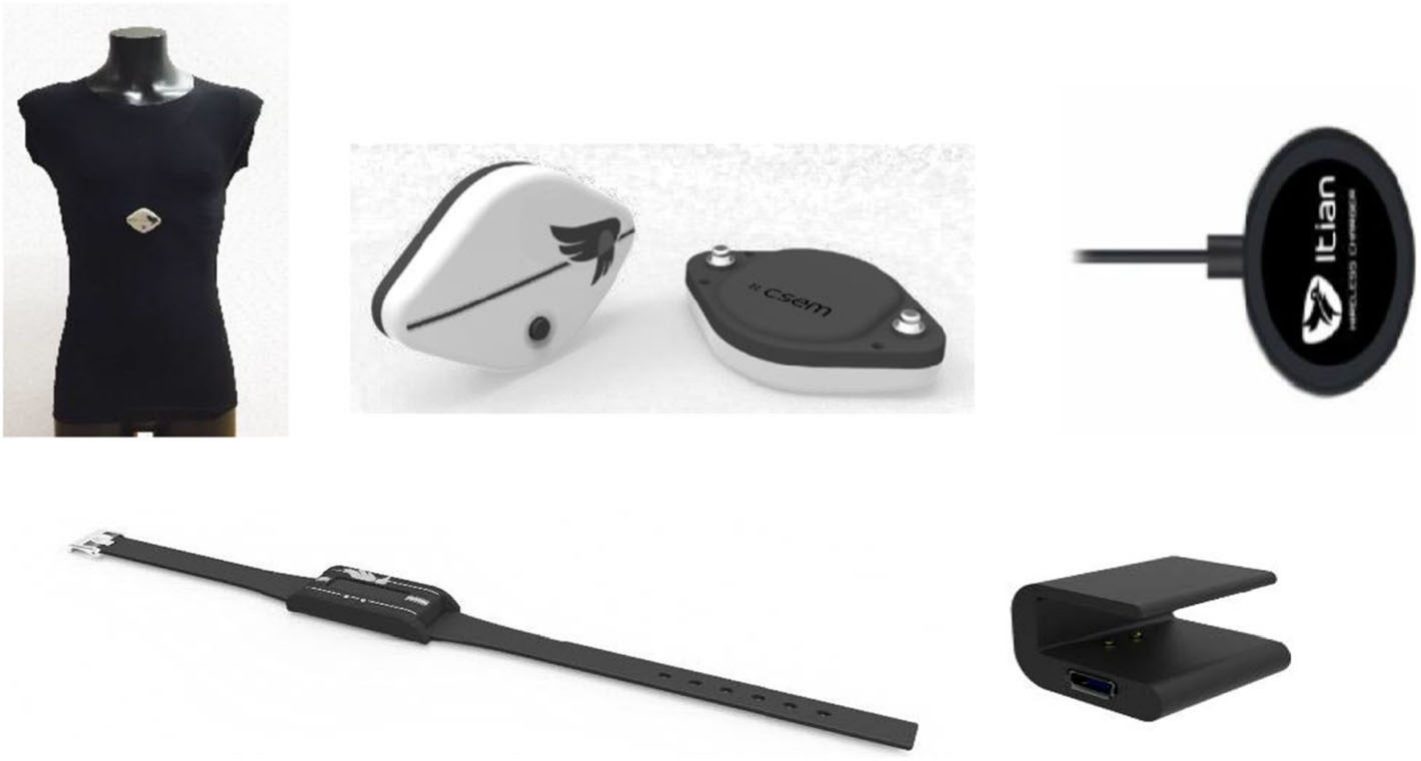
This figure shows PEGASO smart garment and a wrist-worn activity tracker

The PEGASO system has two sets of physical activity monitors; a smart garment and a wrist-worn activity tracker (Fig. 1). Participants are asked to wear the smart garments as often as possible in typical environments when being physically active e.g. at school during physical education, after school sports club, outdoors, commuting to school, while exercising, at home and sleeping. The smart garment is a T-shirt/cropped top with embedded textile electrodes and a monitoring device (data logger). The data logger attaches onto the front side of the T-shirt by using standard snap buttons. The smart garments measure heart rate, physical activity levels, and postures [31]. The activity bracelet monitors physical activities and sleep and participants are asked to wear the device daily during day and night, while removing it for swimming, showering and bathing. Data collected through the smart garment and bracelet is transferred to the smart phone and linked to the Companion App, Dashboard App and the serious game.

#### Web portal

The web portal allows participants to socially interact with each other and to access tutorials on how to improve their diet and increase their physical activity levels through educational training modules. The web portal is accessible from both mobile devices and computers.

#### Companion app

**Fig. 2:**
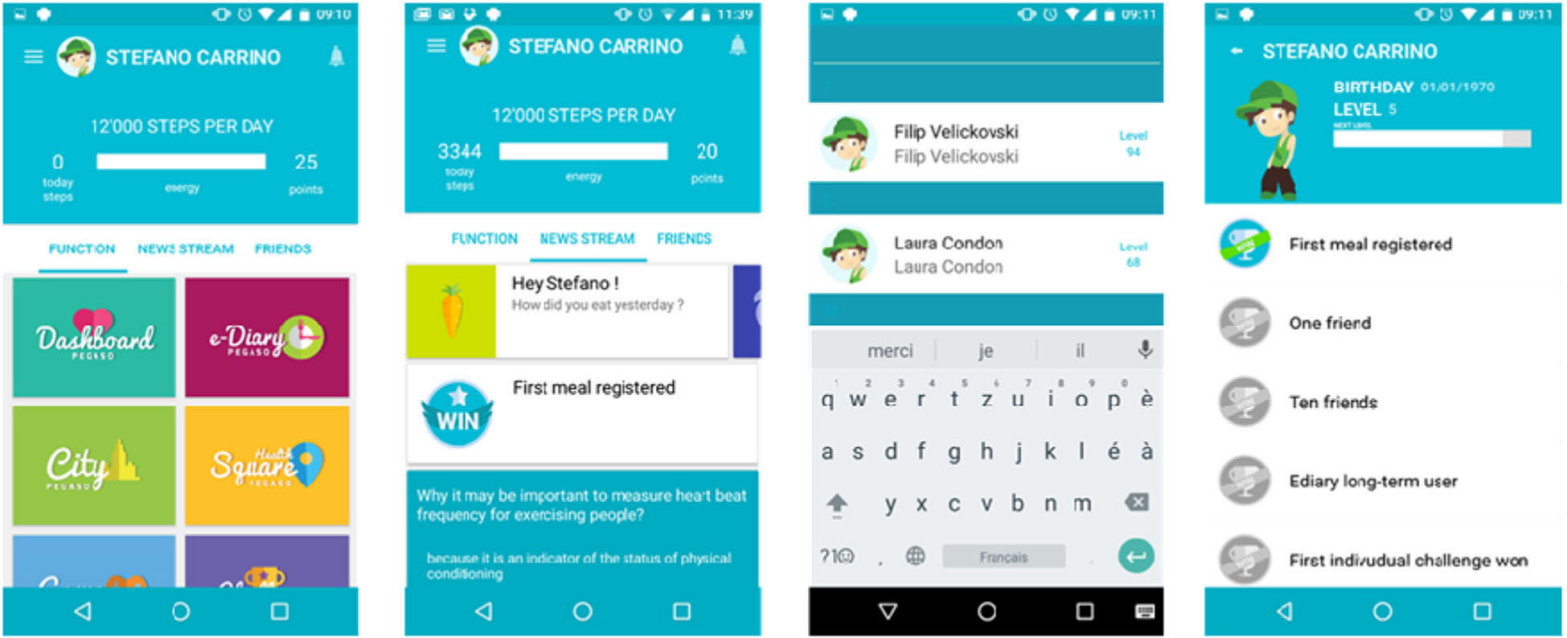
This figure shows some screen shots of the Companion application

The objective of the Companion app is to encompass all other PEGASO apps (Fig. 2). The Companion App aims to act as a personal digital friend which coaches, cares, and empowers participants in developing healthy habits. It aims to provide the participant with a dynamic stream of educational and motivational messages and updates about their achievements and their friends’ shared achievements. Data acquired via physical activity monitors is presented to the participant via a specific interface presented within the Companion app. The Companion app also includes a reward system and the gamification module to allow the participants to: a) achieve one time rewards (e.g., FitCoins, badges, experience points) for the selected target behaviour and b) achieve cyclic events.

#### Challenges app

Through the Challenges app participants can create and accept different types of challenges: individual, competitive or collaborative challenges related to the target behaviours (Table 2). Participants are asked to engage in healthy eating and/or physical activity behaviour to meet the selected challenge. Participants gain points when a challenge is successfully completed (e.g. engaging in 30 min of moderate to vigorous physical activity).

#### EDiary app

**Fig. 3:**
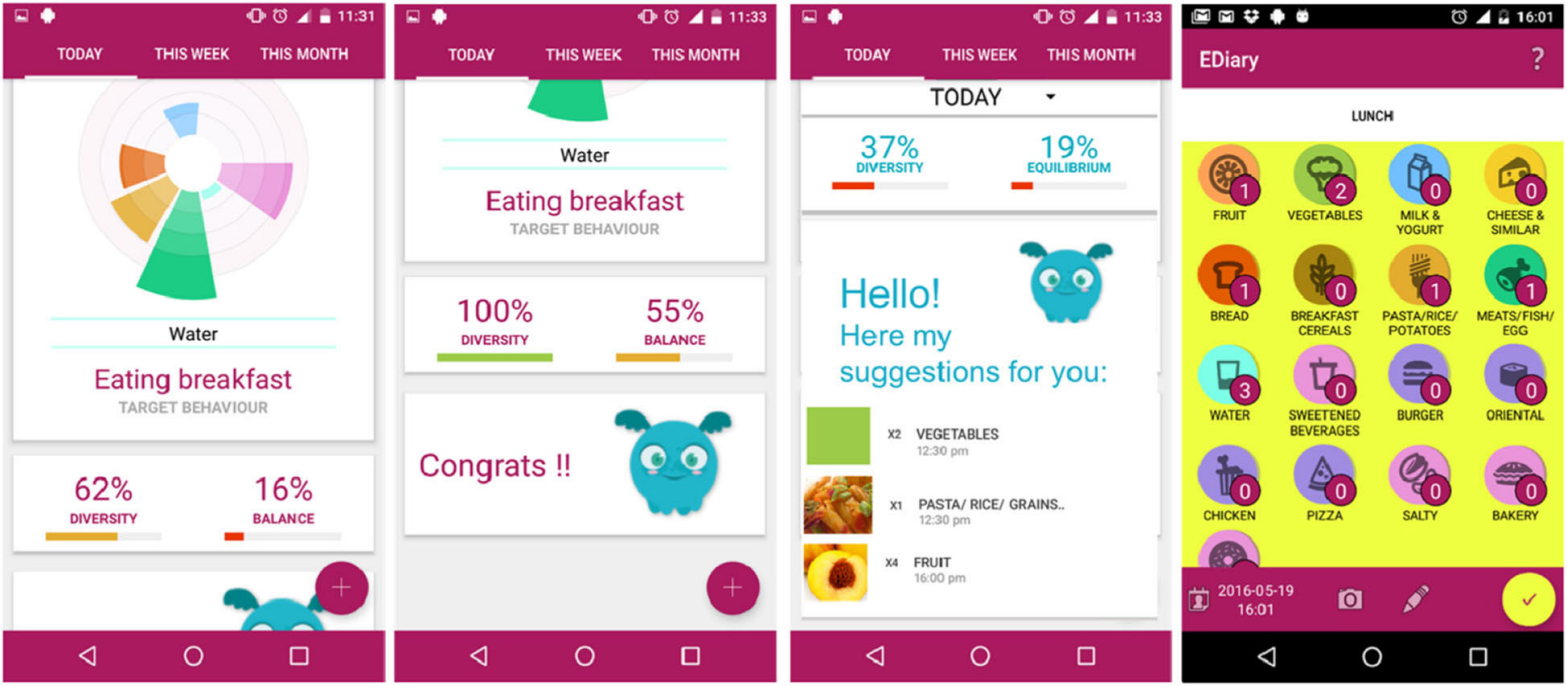
This figure shows some screen shot of the EDiary application

This app allows the participant to enter information about their diet and provides feedback about the balance and diversity of consumed food (Fig. 3). It aims to provide guidance on recommended food groups for the next meal based on participants’ behaviour. The EDiary app is gamified aiming to improve the participants’ experience through introducing an element of fun that can sustain engagement within the app and daily eating habits.

#### Dashboard app

This app allows participants to self-monitor their sleep time and physical activity behaviours: number of steps and minutes spent in moderate-to-vigorous physical activity (MVPA), type of activities made during the course of the day. Activities requiring energy expenditure above 4 METs (as estimated from outputs of the sensor devices) were considered as MVPA, which allows for the higher resting metabolic rate of adolescents [32].

#### Serious game

The serious game aims to serve a central role as the motivational component of PEGASO. As such, it needs to entertain and engage the player, whilst utilising the PEGASO system to capture information about lifestyles and provide relevant feedback to encourage positive health behaviour changes. There are two central behavioural mechanisms within the game: 1) An “energy bar”, used up by the player’s actions in the game, and replenished when achieving their behavioural goals such as increased physical activity or improved diet, and 2) the game implements “research” mechanics that require the player to apply and develop their nutritional knowledge of various food sources.

#### Report app

Although not being tested in the present study, PEGASO consortium developed this app, which aims to connect the PEGASO system with the healthcare system in counties with electronic health records supporting user data sharing (in this study Italy and Spain). Through a bridge *system* within the Report app, it is planned that data collected by the PEGASO system will be extracted and shared with the health professional in the form of a summary report which will be added to the personal health folder.

### Procedures

Eligible participants are introduced to the PEGASO system after they and their parents/guardian provided written and signed consent, and after baseline measurements are collected. Participants are provided with a smart phone (running as OS Android, version from 4.4 to 6.0) with the PEGASO apps installed for the duration of the study.

Given the complexity of the PEGASO system, the components are introduced in two sessions within the same first week. The physical activity monitors are introduced to the participants at the mid-point assessment time at month 4 of the study. This time point was chosen to reduce the burden on the schools and participants, allocating time and resources in addition to the baseline measures, mid-point and follow-up assessment contact points, whilst additionally avoiding conflict with exam timetables and extended holiday periods (e.g. summer break). In addition to familiarising the participants with the functionalities and use of the garment and activity monitors, researchers assist participants in connecting the activity monitors with individual smart phones. All introduction sessions across the four study sites provide a mixture of interactive workshops, educational games, and replicate the method as much as possible within the constraints of the school requirements in different countries.

Participants are offered support during the study to facilitate understanding of how to use the PEGASO system and to trouble-shoot any issues arising. Participants are invited to join a site specific Google Hangout group to allow timely communication with the field researchers who provide solutions to technical issues whenever possible. Teachers at schools are offered a brief training session to be able to answer simple questions related to the use of the PEGASO system. They are asked to direct the participants to the web portal and the FAQ link for questions they cannot answer.

The role of teachers is to support students over the testing period, and to facilitate the communication flow among field researchers, parents and teenagers. During on-site contact points an interaction with teachers is expected; they will have the opportunity to indicate to the field researchers any comments, doubts or conflicts including maladaptive behaviours.

### Comparison group

Schools and participants of the comparison group are given information about the purpose of the study; excluding details about the PEGASO system, at a single face-to-face session held by the field researchers. The comparison schools and participants are asked to continue their routine daily physical and educational activities related to leading a healthy lifestyle.

### Data collection and security

Data are directly recorded by the platform or on-line questionnaires and stored in the Amazon Cloud. The confidentiality of collected data is ensured in accordance with the provision of European current legislation on personal data protection: 95/46/EC EU Directive (DPD) on Data Protection [33] and the EU 2016/680 General Data Protection Regulation [34].

### Outcomes and variables

The first block of outcomes that will be assessed are related to recruitment and adherence to the intervention, namely recruitment rate, study retention, adherence to the intervention and attitudes to the different components of the intervention.

We aim to test a range of outcomes related to individual lifestyle (dietary habits, physical activity, sedentary behaviour, and sleeping habits), knowledge/awareness about these health habits and user experience of the platform. Individual lifestyle and knowledge/awareness about health habits are assessed at baseline and at the end (6 months) of the intervention both in the comparison and intervention group. User experience is assessed at month 2, 4 and 6 only in the intervention group. Outcomes, instruments and assessment points during the study are detailed in Table 3.

**Table 3:** Outcomes, instruments and assessment points in the PEGASO study

Outcome	Instrument	Assessment point			
		BL	M2	M4	M6
Dietary habits	Mediterranean Diet Quality Index in children and adolescents (KIDMED questionnaire) + 5 additional questions	X			X
Physical activity	Physical Activity Questionnaire for Adolescents (PAQ-A) + 3 additional questionnaires	X			X
Sedentary behaviour	Screen time-based sedentary behavior questionnaire (SSBQ)	X			X
Sleeping habits	Sleep Habits Survey (SHS)	X			X
Knowledge questionnaire	Ad hoc questionnaire	X			X
Anthropometric Measurements		X			X
Height	Stadiometer SECA 213	X			X
WC	Flexible graduated measuring tape (SECA 201)	X			X
Weight	*England, Catalonia, Italy*: Homologated	X			X
BMI	electronic scale Tanita SC240MA				
% of body fat	*Scotland*: Tanita MC780MA Multi	X			X
% of body water	Frequency Segmental Body Composition Analyser	X			X
Family affluence and self-reported health status	Family Affluence Scale (FAS) + 1 question from the Social Functioning subscale −12 (SF-12)	X			
*Motivation*	*Perceived Competence Scale*	*X*	*X*	*X*	*X*
*Usability*	*System Usability Scale*			*X*	*X*
*Emotional Response to the Platform*	*Emotional Metric Outcome questionnaire*			*X*	*X*
*Satisfaction, Trust, Reputation*	Ad hoc *questionnaire*			*X*	*X*
*User Experience*	*Focus Groups*			*X*	*X*

In italics, outcomes assessd only in the intervention group. All the other outcomes were assessed in both, intervention and comparative group

Additional questions: Research team included additional questions in order to better assess the target outcome in case no validated questionnaire were found

Parent’s undertook an ad-hoc questionanire in order to assess sociodemographic characteristics, Socio Economic Status (SES) and Family structure and living conditions of the family

*WC* Waist Circumference, *BMI* Body Mass Index

*BL* Baseline, *M2* Month 2, *M4* Month 4, *M6* Month6

Individual lifestyle outcomes are measured through on-line questionnaires. Dietary habits are assessed through the KIDMED, a 16 item semi-quantitative food habits questionnaire which aims to assess dietary patterns among children, adolescents and young adults [35]. Additional questions are also included to assess the consumption of fast food and sugar-sweetened beverages, breakfast skipping and snacking behaviour. Physical activity is assessed through the Physical Activity Questionnaire for Adolescents (PAQ-A) [36]. Additional questions and an extra question from PAQ-elementary are included to assess those issues related to PA not covered by the PAQ-A such as transportation to school. Sedentary behaviour is assessed by the screen time-based sedentary behaviour questionnaire (SSBQ) from the Healthy Lifestyle in Europe by Nutrition in Adolescence study (HELENA study), which has been validated among participants in the HELENA study against the use of an accelerometer. Participants are asked to report their habitual time devoted to several sedentary behaviours, mostly related to screen time: (a) TV viewing, (b) computer games, (c) console (video) games, (d) Internet for non-study reasons (hobbies), (e) Internet for study reasons and (f) study time (out of scholar schedule) [37]. Sleeping habits are assessed through four questions about sleep duration from the Sleep Habits Survey for Adolescents (SHSA), a validated self-reported survey that estimates sleep patterns in adolescents [38].

Knowledge/awareness about health habits are assessed through an ad-hoc questionnaire (quiz format) that covers knowledge of dietary habits, physical activity and sleeping behaviour. Most of the questions are from a questionnaire, which is validated in Italy [39] and some others are specifically created.

Motivation to maintain or increase healthy dietary habits and physical activity will be assessed through the “Perceived Competence Scale” a short 4-item scale that assesses the degree to which participants feel confident about being able to make (or maintain) a change toward a healthy behaviour [40]. Adolescents’ age, gender, perceived health status level and family affluence will be included in the on-line questionnaires. Parent’s (or legal guardian’s) educational level, occupation and familiar structure will be also documented only at baseline.

User experience (UX) refers to a person’s total experience using a particular product, system or service [41]. The factors of UX to be investigated during the study include: emotion, engagement, trust, reputation, pleasure/hedonism, usability, acceptability, accessibility, connectedness, context and target behaviours adherence. The PEGASO study aims to holistically assess how the teenagers experience the system in their everyday lives and where and how this experience facilitates use and where it can be improved. Mixed methods (quantitative and qualitative methodology) will be used to assess UX, specifically through self-reported questionnaires and focus groups or semi-structured interviews, respectively. The System Usability Scale (SUS) is a well established 10 item questionnaire with five response options to assess usability of a variety of products and services, where usability is a distinct concept within the overall understanding of UX [42]. SUS will be applied at month 4 to assess usability of the Companion and the game and at month 6 for the whole system. The Emotional Metric Outcome (EMO) questionnaire is a 16 item questionnaire to assess the emotional outcomes of interaction, both positive and negative [43]. The EMO will be undertaken at month 4 and 6. A questionnaire has been created ad hoc in order to measure the satisfaction, trust and reputation of the platform. This comprises a 28 item questionnaire with 5 answer categories and is undertaken at months 4 and 6.

### Focus groups

Key contributors to understanding the UX of the PEGASO system such as; usability, acceptability, accessibility, context of use, connectedness, desirability and pleasure will also be assessed through focus groups; moreover they will be used to gain insight on users understanding of the system and their experience and motivation to engage/disengage with the PEGASO system and its different component parts (apps, games and sensors). Participants in the focus groups will be recruited from participants in the intervention group. A total of four focus groups per participating site (England, Lombardy and Catalonia) will be undertaken at month 4 and 6. Participants in focus groups at month 4 will have only tested the apps and the game of Pegaso, whilst participants on the Month 6 focus groups additionally will have used the sensors for 2 months. Each focus group will consist of 4–8 participants. The groups, whenever possible, will be gender-balanced with different levels of engagement to the PEGASO system. A trained moderator will guide the focus group following a script and will be assisted by an observer. All of them will be audio and/or videotaped and transcribed systematically, literally, and anonymously. An analysis will be made of thematic content, coding the data and grouping them into predefined categories based on topics.

### Anthropometric variables

Anthropometric variables are assessed at baseline and at the end of the intervention as secondary outcomes. Body weight and percentage of body fat is assessed by bioelectric impedance analysis (BIA) performed using a homologated electronic scale (Tanita sc-240 ma) with the participant wearing light clothing and no shoes. Height is also measured with a portable system with the participant shoeless in the standing position (SECA 213). Body mass index (BMI) is calculated as weight (kg) divided by height squared (m2). Waist circumference is measured using a flexible graduated measuring tape (SECA 201) with the patient in the standing position without clothing covering the midsection of the torso. All the anthropometric measurements are undertaken in the most private way possible.

### Data collected by the PEGASO system

The interaction between the user and the Pegaso system is recognized through of a set of automatic engagement indicators measuring the actual usage of the system. For each participant, actions like interacting with the Companion app or registering a meal in the EDiary app are tracked and daily stored. The EDiary app records information about the reported meals composition, food groups and servings. The smartphone collects information about physical activity such as the number of steps or the total time spent doing physical activity. Further information on physical activity is collected from the 4th month by the introduction of a smart bracelet and smart sensor. General information about healthy habits is gathered through the “Self-Report Habit Index (SRHI)” adapted to include items that reflect self-identity [44]. The questionnaire appears as a notification inside the Companion app in between the achievement of a target behaviour and the selection of a new one. Self-awareness is recorded by the PEGASO system using the COM-B-Q Self-Assessment Questionnaire (Capability, Opportunity and Motivation for Behaviour) [45]. It is a questionnaire, based on the principles of behaviour change, about current behaviours and insight into aspects that need to be acted upon in order to achieve a pre-specified behavioural goal. The questionnaire appears as a notification inside the Companion app just after the selection of a new target behaviour.

### Analysis plan and statistical analysis

The analysis plan will include a description of all data at every assessment point and an overall comparison of baseline characteristics between intervention group (IG) and comparison group (CG), as well as by sites and gender. Knowledge/awareness, lifestyles and anthropometric measurements will be compared at pre and post intervention (month 6) utilising an intention-to-treat (ITT) approach, independently from drop-outs and non-adherence. Relevant changes among lifestyles and knowledge will be defined according to current recommendations from international guidelines [46]. The effect of the intervention could be modified by gender, cultural and socioeconomic level, body mass index (BMI), as well as by the baseline lifestyles, which will be controlled in the analysis. Changes in motivation will be assessed in the four assessment points and compared to baseline. For the intervention group, the results from SUS, EMO, satisfaction, trust and reputation questionnaires will be compared from month 4 to month 6. The level of engagement in the PEGASO platform will be assessed from indicators of usage at individual level, such as the number of days of interactions with the APPs or the daily number of steps, stored in the cloud.

Results will be reported as mean ± standard deviation (or median, percentile 25 and 75) for quantitative variables or frequency and percentage distribution for qualitative variables. Quantitative variables will be tested for normality, log-transformed if needed, and accordingly analysed with parametric or non-parametric methods. Chi-squared test will be used to analyse the association between independent qualitative variables, along with the McNemar test for paired samples. The Student t-test will be used for the comparison of means between two groups, and the paired t-test will be applied to assess changes within one same group. Alternatively, the corresponding nonparametric tests will be used, as required. The relationship between quantitative variables will be analysed using Pearson’s correlation coefficient or Spearman coefficient in the case of asymmetrically distributed variables. Techniques of principal component analysis (PCA) will be applied to identify patterns of variables best explaining outcomes of interest.

Multivariate linear regression analysis and logistic regression analysis will be used to analyse the variables determining the changes in physical activity and eating and sleeping habits. In order to analyse the effect of the intervention, comparison will be made of the changes observed in the CG versus the IG, with estimation of the Cohen d statistic, adjusting for the variables that may influence the results.

The level of statistical significance will be set at α = 0.05 and all tests will be two-tailed. Statistical analyses will be carried out using the software packages SPSS, version 22.0 (SPSS Inc., Chicago, IL) and Stata (StataCorp. 2009. Stata Statistical Software: Release 11. College Station, TX: StataCorp LP). Focus groups will analysis will be done with the support of Atlas. Ti /NVIVO and by triangulation of analyst.

## Discussion

In general, and in the field of health and medicine in particular, in recent years information and communication technologies have experienced rapid growth. Some authors point out that the use of the internet and associated technologies such as computer apps represent a revolution within the medical field, since they are fast, versatile, manageable, illustrative tools that allow people to be empowered with their health [47]. However, this vast development of health apps and those related with healthy lifestyles promotion are not encompassed by clear and sufficient scientific evidence on health improvement. For instance, two recent systematic reviews concluded that mobile phone interventions can diminish sleep disorders and to enhance sleep quality [48] and mobile phone app-based interventions may be useful tools for weight loss [49]. However, some other reviews showed modest evidence on the efficacy of intervention based on health apps on the promotion of healthy habits related with diet, physical activity and sedentary behaviour in children and adults [50] or in improving preventive behaviours among adolescents [51, 52]. Schoeppe and collaborators concluded in their systematic reviews that multi-component interventions appear to be more effective than stand-alone app interventions [50]. In this sense, the PEGASO system could be considered as multi-component intervention, which includes several apps, a game and smart sensors, and which targets multiple health behaviours All the reviews highlighted the necessity to a) involve end-users on the development of app-based interventions and b) evaluate the health effects of app-based interventions by conducting experimental studies to obtain sufficient scientific evidence [48–51].

The first part of the PEGASO F4F study developed a multi-dimensional ICT based intervention that includes the use of smartphone applications, a game and the use of wearables to promote healthy habits among adolescents. An iterative pre- trial study has already been undertaken to achieve the user centred design approach of PEGASO F4F. The aim of this was to increase the likelihood that the system will meet the requirements of teenagers and be accepted by this specific group of end users. Volunteers from the 4 sites tested the platform in different moments of the development in an iterative process. Their feedback was used to assess user interaction and experience, usability and the security and confidentiality requirements of the PEGASO system.

The present study aims to test the methodology to investigate the effectiveness that the use of the Pegaso system will have on teenage health awareness and the improvement of healthy habits with regards to adherence to recommendations and habit formation in terms of dietary behaviour, physical activity and sedentary and sleeping behaviour among adolescents.

Moreover, the present study will assess the user experience through validated questionnaires and focus groups in order to receive feedback from end users when they are introduced to the new technology, over a short-term period of use and then also over a longer period of time, 6 months after set up. The purpose of this is to understand how teenagers utilize proactive mHealth systems over time, understand the relationship between UX and health behaviours/ habit formation, and provide points of learning for industry looking to develop health ICT’s and other interventions for young people; specifically adolescents.

The study follows most of the recommendations of the CONSORT for quasi experimental studies [53]; however, participants are not be blinded to the intervention due to the nature of the intervention. Self-reported data is used to measure main results related to healthy habits modification and knowledge. These are assessed through the validated questionnaires selected for this trial.

According to the availability of each site, two approaches for the selection of comparative groups were foreseen; the comparative group either came from the same school or from a different school. Both, clustered and non-clustered quasi-experimental studies have their own advantages and disadvantages. A possible cross-over effects regarding the fact that both intervention and comparative groups are from the same schools may occur; social peer influence can be strongly associated to lifestyles and influence the results of the comparative group. However, we believe that adolescents tend to mainly interrelate with mates from their same class. Moreover, PEGASO F4F is not a school-based intervention itself; on the contrary, it was conceived as an individual intervention with several social components. By having both approaches it can provide us with some insight regarding the role of the social component and the impact if may have on the intervention.

The results of this study target improvements on dietary habits, physical activity and sedentary time and sleep quality among adolescents. Specifically results could lead to:

- improvement in adolescent lifestyles (in terms of awareness, adherence and habit formation)
- improved knowledge among adolescents about health and wellbeing and the impact of their personal day to day decisions on their health
- an appreciation of how new technologies based on mHealth can assist them in their lifestyle choices and promote positive health behaviours.
- Improved teenage mHealth interventions that are more appropriately designed to meet the specific needs of these young users

Positive results from this ICT based multi component intervention may represent an opportunity and influential tool against the development of chronic disease during adulthood.

## Data Availability

Not applicable.

## Abbreviations

App: Informatics application
BMI: Body Mass Index
EMO: Emotional Metric Outcomes
ICT: Information and Communication Technologies
mHealth: Mobile Health
PAQ-A: Physical Activity Questionnaire for Adolescents
PEGASO: Personalised Guidance Services for Optimising lifestyle in teen-agers through awareness, motivation and engagement
SHS: Sleep Habits Survey
SSBQ: Screen time-based sedentary behaviour questionnaire
SUS: System Usability Scale
UX: User Experience
