# Correction to: Promoting healthy teenage behaviour across three European countries through the use of a novel smartphone technology platform, PEGASO fit for future: study protocol of a quasi-experimental, controlled, multi-Centre trial

**DOI:** 10.1186/s12911-021-01686-5

**Published:** 2021-11-30

**Authors:** Elisa Puigdomenech, Anne Martin, Alexandra Lang, Fulvio Adorni, Santiago Felipe Gomez, Brian McKinstry, Federica Prinelli, Laura Condon, Rajeeb Rashid, Maurizio Caon, Sarah Atkinson, Claudio L. Lafortuna, Valentina Ciociola, Janet Hanley, Lucy McCloughan, Conxa Castell, Mireia Espallargues, the PEGASO Fit For Future Consortium

**Affiliations:** 1 Agency for Health Quality and Assessment of Catalonia (AQuAS), Catalan Department of Health, Roc Boronat 81-95, 2nd floor, 08005 Barcelona, Spain; 2 Health Services Research on Chronic Patients Network (REDISSEC), Instituto de Salud Carlos III, Roc Boronat 81-95, 2nd floor, 08005 Barcelona, Spain; 3 MRC/CSO Social and Public Health Sciences Unit, University of Glasgow, 200 Renfield Street, Glasgow, G2 3AX UK; 4 Human Factors Research Group, Faculty of Engineering, University of Nottingham, University Park, Nottingham, NG7 2RD UK; 5 National Research Council, Institute of Biomedical Technologies, Via Fratelli Cervi, 93, 20090 Segrate, MI Italy; 6 Programs Department Gasol Foundation, 26-28 Jaume I street, 08830 Sant Boi de Llobregat, Spain; 7 GREpS. Health Education Research Group, Nursing and Phisiotherapy Department, University of Lleida, 2 Montserrat Roig street, 25198 Lleida, Spain; 8 Usher Institute, University of Edinburgh, NINE Edinburgh BioQuarter, 9 Little France Road, Edinburgh, EH16 4UX UK; 9 PRISM Research Group, Division of Primary Care, School of Medicine, University of Nottingham, Room 1404, Tower Building, University Park, Nottingham, NG7 2RD UK; 10 Deanery of Clinical Sciences, College of Medicine and Veterinary Medicine, The Queen’s Medical Research Institute, University of Edinburgh, Edinburgh BioQuarter, 47 Little France Crescent, Edinburgh, EH16 4TJ UK; 11 College of Engineering and School of Management, University of Applied Sciences and Arts Western Switzerland (HES-SO), chemin du musée 4n, 1700 Fribourg, Switzerland; 12 Human Factors Research Group, Faculty of Engineering, The University of Nottingham, University Park, Nottingham, NG7 2RD UK; 13 Consiglio Nazionale delle Ricerche, Istituto di Fisiologia Clinica, Piazza Ospedale Maggiore, 3, 20162 Milano, Italy; 14 School of Health and Social Care, Edinburgh Napier University, Sighthill Court, Edinburgh, EH41 3ND UK; 15 Usher Institute, University of Edinburgh, NINE Edinburgh BioQuarter, 9 Little France Road, Edinburgh, EH16 4UX UK; 16 Catalonia Public Health Agency, Catalan Department of Health, Roc Boronat 81-95, 3rd floor, 08005 Barcelona, Spain

## Correction to: BMC Med Inform Decis Mak (2019) 19:278 10.1186/s12911-019-0958-x

Following publication of the original article [1], the authors reported a missing statement in the Funding declaration. The updated Funding declaration is given below with the inserted statement in boldface:

This project was funded by research grants of the European Commission under the 7th Framework Programme (Call identifier: FP7-ICT-2013-10; Project number: 610727) and of the **Instituto de Salud Carlos III through the project RD16/0001/0010 (Co-funded by European Regional Development Fund/European Social Fund "A way to make Europe"/"Investing in your future")**. Funding has allowed the evaluation work package of the project to design the study, collect and analyse data and in writing the manuscript. AM was supported by the UK Medical Research Council (grant number MC_UU_12017/14) and the Scottish Government Chief Scientist Office (grant number SPHSU14).

The original article [1] has been updated.
